# Differential Induction of Astaxanthin, Lutein, and Canthaxanthin with Altered Fatty Acid Profiles in *Chromochloris zofingiensis* via a Two-Stage Cultivation Approach Using Different Chemical Modulators

**DOI:** 10.3390/life16050799

**Published:** 2026-05-11

**Authors:** Suthamat Niyompanich, Pokchut Kusolkumbot, Watcharee Kunyalung, Atthaboon Watthammawut, Sorawit Powtongsook

**Affiliations:** 1 Department of Biology, Faculty of Science, Srinakharinwirot University, Bangkok 10110, Thailand; 2 Biodiversity Research Centre, Thailand Institute of Scientific and Technological Research (TISTR), Pathum Thani 12120, Thailand; pokchut@tistr.or.th (P.K.); watcharee@tistr.or.th (W.K.); 3 Department of Anatomy, Faculty of Medicine, Srinakharinwirot University, Bangkok 10110, Thailand; atthaboon@g.swu.ac.th; 4 National Center for Genetic Engineering and Biotechnology, National Science and Technology Development Agency, Pathum Thani 12120, Thailand; sorawit@biotec.or.th; 5 Center of Excellence for Marine Biotechnology, Department of Marine Science, Faculty of Science, Chulalongkorn University, Bangkok 10330, Thailand

**Keywords:** *Chromochloris zofingiensis*, chemical modulators, astaxanthin, lutein, canthaxanthin, fatty acids, two-stage cultivation approach

## Abstract

*Chromochloris zofingiensis* is a promising source of high-value bioproducts, particularly carotenoids and fatty acids. In this study, three selected chemical agents, including methylene blue (MB), salicylic acid (SA), and zinc sulfate heptahydrate (ZN), representing their roles as an oxidant, a signal transducer, and a metal ion, respectively, were applied at 96 h post-inoculation to stimulate metabolite accumulation via a two-stage cultivation approach. None of the treatments significantly affected algal growth. Among the treatments, HPLC analysis showed that 2.5 mM ZN significantly exhibited a dual stimulatory effect on astaxanthin (1.679 ± 0.122 mg g^−1^) and lutein (4.257 ± 0.183 mg g^−1^) accumulation, which were 2.28- and 2.91-fold higher than the control, respectively. The 1 µM MB significantly enhanced the canthaxanthin content to 2.382 ± 0.210 mg g^−1^ (a 3.57-fold increase). Different SA concentrations selectively induced the target pigments of astaxanthin and lutein. APCI-QTOF analysis enabled the detection of echinenone in the microalgal extracts. Its identity and quantification were subsequently validated by HPLC, with the highest content detected under the 0.2 mM SA treatment. GC-FID analysis revealed changes in the composition of six major fatty acids, with C18:1 n-9 representing 50.01% of the total fatty acids under the 2.5 mM ZN treatment. These findings suggest that the two-stage approach could offer a practical and feasible strategy for microalgal biorefineries.

## 1. Introduction

The microalga *Chromochloris zofingiensis* has emerged as a promising cell factory for its capacity to synthesize a diverse range of high-value bioactive products, such as carotenoids, lipids, and exopolysaccharides [1,2]. The major carotenoids, including astaxanthin, lutein, and canthaxanthin, are found in *C. zofingiensis* [1,2]. These compounds offer strong antioxidant activities and have beneficial effects on human and animal health by promoting protective actions against several diseases caused by oxidative damage [2,3,4], thus highlighting their importance in the nutraceutical and pharmaceutical industries. Moreover, *C. zofingiensis* is able to grow in diverse cultivation modes, including photoautotrophic, heterotrophic, and mixotrophic conditions and allows for efficient scaling-up for both indoor and outdoor production. *C. zofingiensis* is also a potent source of lipids as it can accumulate high contents of triacylglycerols (TAGs) up to 40% of dry weight [1], making it an attractive feedstock for biodiesel production. Typically, biodiesel is composed of fatty acid methyl esters (FAMEs) that are synthesized via the transesterification of oils or fats [5]. Thus, investigating the composition of fatty acids, including saturated and polyunsaturated fatty acids that constitute the main components of TAGs, is crucial for determining the key properties of biodiesel [6]. In addition to biofuel applications, algal fatty acids, such as palmitic acid (C16:0), oleic acid (C18:1), and omega-3 and omega-6 fatty acids (such as linoleic acid (C18:2), α-linolenic acid (C18:3 n-3), and gamma-linolenic acid (C18:3 n-6)), provide high market values that can be potentially commercialized for the nutraceutical, cosmetic, and food industries [7]. These features thus position *C. zofingiensis* as a proficient natural producer, comparable to other microalgal species, serving as a platform for the production of multiple high-value bioproducts in biorefinery applications [1,8].

The accumulation of these high-value microalgal compounds is generally induced by environmental stress conditions. Manipulation of different nutritional and physical stress factors (such as nutrient deprivation, high light intensity, and high salinity) has been extensively developed and applied to microalgal cultures to increase the biosynthesis of target compounds [9]. Alternatively, the addition of chemical modulators can be employed to enhance bioproduct accumulation. These agents are required in minute amounts to be supplemented in culture media and effectively function by either inducing an oxidative stress response or modulating specific metabolic pathways or other aspects of cellular mechanisms [10]. Nevertheless, the introduction of stress conditions in microalgal cultures can result in an inhibition of cellular growth, leading to reduced biomass production and a lower accumulation yield of target compounds [11]. Recently, the two-stage cultivation approach in microalgae has been developed as an economic and feasible strategy to fully exploit their microalgal productivity [12]. This approach consists of two sequential stages: the first stage optimizes conditions for maximal biomass production, while the second stage employs stress conditions to induce target compound accumulation [9]. In the present study, two-stage cultivation was employed in *C. zofingiensis* to achieve high microalgal biomass under a mixotrophic condition in the first stage, prior to applying chemical modulators in the second stage to induce unfavorable conditions in the microalgae. By supplying both light and an organic carbon source, mixotrophic cultivation promotes enhanced biomass accumulation [13] and is thus used to achieve sufficient biomass before chemical induction in the second stage. The focus of this study on carotenoid and fatty acid profiling was based on the data that they exhibit a lipophilic nature (conferred by long aliphatic chains) and may originate from a common precursor (pyruvate) utilized by both the carotenoid (via the MEP pathway) and the fatty acid biosynthetic pathways [14].

In this study, three selected chemical modulators, including methylene blue (MB), salicylic acid (SA), and zinc sulfate heptahydrate (ZnSO_4_·7H_2_O; ZN) were selected and exogenously introduced at various concentrations to *C. zofingiensis* cultures in the second stage of cultivation. MB, SA, and ZN represent distinct chemical roles as an oxidant, a signal transducer, and a metal ion, respectively, and have been previously reported to induce unfavorable environmental conditions and enhance the production of high-value metabolites in diverse microalgae species. MB acts as an oxidant generating singlet oxygen (^1^O_2_), which is a type of reactive oxygen species (ROS), thereby inducing oxidative stress that promotes the accumulation of various target bioproducts, as demonstrated by the enhanced astaxanthin production in *Haematococcus pluvialis* [10,15]. The generated ROS may promote carotenoid formation by directly influencing carotenogenic enzymes, thereby protecting cells against oxidative damage [16]. SA is a signaling molecule that not only mediates plant defense responses to environmental stressors but also plays a role in modulating plant growth and development [17]. Moreover, SA has previously been shown to induce oxidative stress in *H. pluvialis* by affecting key antioxidant enzymes (SOD, CAT, and APX), and SA is suggested to act as a stress modulator that promotes the biosynthesis of secondary carotenoids [18]. ZN is generally used as an algal culture media compositions [19]. The Zn^2+^ metal ion can serve as an essential factor providing significant functions tomicroalgal enzymes (such as superoxide dismutase and carbonic anhydrase) involved in antioxidant systems and carbon concentrating mechanism (CCM)-driven fatty acid biosynthesis [20,21]. Nevertheless, the effects of these chemical modulators in combination with the two-stage cultivation approach on the microalgal growth and profiling of carotenoids and fatty acids in mixotrophic *C. zofingiensis* remained largely unexplored. Therefore, the use of the selected three chemicals with different modes of action may allow to explore their roles in modulating physiological and metabolic responses associated with changes in the carotenoid and fatty acid profiles in *C. zofingiensis*. Information on the major carotenoid and fatty acid compositions in response to chemical treatments was elucidated using HPLC, APCI-QTOF MS/MS and GC-FID analyses. The knowledge gained from this study should be informative for the future improvement of the efficient production of the desired high-value compounds in *C. zofingiensis* through the use of exogenous chemical modulators and eventually for large-scale cultivation in various venues of industrial applications.

## 2. Materials and Methods

### 2.1. Chemicals

All chemicals used for the preparation of the proteose algal medium, including NaNO_3_ (Ajax Finechem, Seven Hills, NSW, Australia), CaCl_2_·2H_2_O (Merck KGaA, Darmstadt, Germany), MgSO_4_·7H_2_O (Fisher Scientific Company, Fair Lawn, NJ, USA), K_2_HPO_4_ (Ajax Finechem), KH_2_PO_4_ (Ajax Finechem), NaCl (Applichem Panreac ITW Companies, Darmstadt, Germany), and proteose peptone (HiMedia Laboratories, Mumbai, India), were of analytical grade. The D-glucose supplemented in the algal medium for batch cultures in mixotrophic conditions was obtained from Fischer Scientific U.K., Limited (Loughborough, Leics, UK). The analytical-grade chemical modulators including methylene blue (MB) and salicylic acid (SA) were obtained from Merck KGaA and Sigma-Aldrich (St. Louis, MO, USA), respectively, and zinc sulfate heptahydrate (ZnSO_4_·7H_2_O; ZN) was from Ajax Finechem. All organic solvents used for carotenoid and fatty acid analyses were HPLC-grade and obtained from Merck KGaA and Fisher Scientific Company. The carotenoid standards including astaxanthin, lutein, canthaxanthin, and echinenone were purchased from Sigma-Aldrich. The standard Supelco 37 Component FAME Mix was purchased from Sigma-Aldrich. The internal standard for fatty acid analysis was heptadecanoic acid (Sigma-Aldrich).

### 2.2. Microalgal Cultivation and Chemical Treatment

*Chromochloris zofingiensis* UTEX 32 was obtained from the University of Texas Culture Collection of Algae (UTEX, Austin, TX, USA). The strain was maintained on agar slants containing the proteose medium, consisting of (per liter): 2.94 mM NaNO_3_, 0.17 mM CaCl_2_·2H_2_O, 0.30 mM MgSO_4_·7H_2_O, 0.43 mM K_2_HPO_4_, 1.29 mM KH_2_PO_4_, 0.43 mM NaCl, and 1 g of proteose peptone. *C. zofingiensis* from the agar slants was inoculated into 10 mL of proteose broth and allowed to grow for 4 days at 25 °C with shaking at 150 rpm and illuminated with continuous fluorescent light of 90 µmol m^−2^ s^−1^ on the flask surface. These cells were then inoculated at a 5% inoculum into a 250 mL Erlenmeyer flask containing 50 mL of the proteose medium, and were grown for 4 days and served as seed cells, as previously described [22].

For batch cultures in mixotrophic conditions, the seed cells were inoculated into 100 mL proteose medium containing 20 g L^−1^ of glucose in a 250 mL Erlenmeyer flask at an inoculum size of 10% (*v*/*v*, with average cell concentration of 0.3 g L^−1^ dry weight). The algal cells were cultured at 25 °C with shaking at 150 rpm, under continuous fluorescent light of 90 µmol m^−2^ s^−1^ on the flask surface. Our algal batch cultures employed a two-stage cultivation strategy to achieve high cell densities under mixotrophic conditions in the first stage, and the subsequent stage involved the supplementation of specific chemical modulators for further inducing stress conditions and enhancing target compound production in *C. zofingiensis*. The three chemical modulators, MB, SA, and ZN, were selected for algal treatment to determine their effects on carotenoid and fatty acid profiles. MB, SA, and ZN were dissolved in sterile distilled water and filtered through a 0.2 µm PES syringe filter. Each of the chemicals was added to the *C. zofingiensis* cultures when cells reached the early stationary phase at 96 h post-inoculation, achieving final concentrations of MB at 0.01–1 µM, SA at 0.1–0.4 mM, and ZN at 2.5–10 mM. The concentration ranges applied in the present study were defined based on previous reports [15,17,23,24] and preliminary screening under our laboratory conditions to achieve metabolite induction without severe growth inhibition. Control cultures without chemical modulators were prepared by replacing the chemical treatments with an equivalent volume of sterile distilled water. All cultures were incubated for a total of 8 days prior to subsequent analysis, and all experiments were conducted in biological triplicate.

### 2.3. Determination of Algal Growth Profiles

Biomass concentration was determined by centrifuging 10 mL of algal cells at 4500× *g* for 5 min and washing the pellet 3 times with distilled water. The collected cells were dried in an oven at 70 °C (until a constant weight was reached) and cooled down to room temperature in a desiccator before weighing [25]. The algal growth curves of the control and treated cultures (MB, SA, and ZN) were monitored at OD680 every two days using a UV-1800 spectrophotometer (Shimadzu Scientific Instruments, Kyoto, Japan). The specific growth rate (µ) at the exponential growth phase was calculated according to the following equation:

Specific growth rate (h^−1^) = (ln X_2_ − ln X_1_)/(t_2_ − t_1_) (1)

where X_1_ and X_2_ are the OD680 values at time t_1_ and t_2_, respectively.

### 2.4. Pigment Extraction and HPLC Analysis

The 8-day-old algal cells were harvested by centrifugation, washed, and lyophilized in a ScanVac Coolsafe 110-4 Pro freeze-dryer (LaboGene ApS, Vassingerød, Denmark). Lyophilized cells (10 mg) were ground in the methanol/dichloromethane (3:1), and extracted repeatedly until the cell pellet became colorless [25]. All extracts were then combined and subjected to centrifugation at 11,000× *g* for 5 min, dried under nitrogen gas, dissolved in 1 mL of extraction solvent, and filtrated through a 0.2 µm PTFE syringe filter prior to HPLC analysis. All processes were performed under dim light. Identification and quantification of the extracted pigments were performed by HPLC analysis, as described previously [25], using an HPLC Shimadzu LC20A (Shimadzu Scientific Instruments, Kyoto, Japan) equipped with the a C18 column (5 µm; 250 × 4.6 mm) (GL Science Inc., Tokyo, Japan) and a diode array detector (DAD), operating at 30 °C. The mobile phase consisted of solvent A (dichloromethane/methanol/acetonitrile/water, 5:85:5.5:4.5, *v*/*v*) and solvent B (dichloromethane/methanol/acetonitrile/water, 25:28:42.5:4.5, *v*/*v*). The HPLC gradient was performed as follows: 0% B for 8 min, a linear gradient of 0–100% B for 6 min, and 100% B for 50 min. The injection volume was 10 µL and the flow rate was 1 mL min^−1^. The carotenoid standards used were astaxanthin, lutein, canthaxanthin, and echinenone. The absorption spectra were detected ranging from 250 to 700 nm and peaks were monitored at 480 nm [25]. The extracted carotenoid pigments were identified by comparing the retention time and maximum absorption spectrum with the four carotenoid standards. The pigment contents of astaxanthin, lutein, and canthaxanthin in each of the *C. zofingiensis* cultures treated with chemical treatments were quantified and compared.

To determine total concentrations of chlorophyll a (C_a_), chlorophyll b (C_b_), and carotenoids, the extracted crude sample was re-dissolved in 1 mL of acetone and the absorbance was subsequently measured at 645, 662, and 470 nm using a UV-1800 spectrophotometer. These pigment concentrations were calculated according to the following formulae, as described by Lichtenthaler, H.K. and Buschmann, C. [26]:

C_a_ (μg mL^−1^) = 11.24 A_662_ − 2.04 A_645_ (2)

C_b_ (μg mL^−1^) = 20.13 A_645_ − 4.19 A_662_ (3)

Total carotenoids (μg mL^−1^) = (1000 A_470_ − 1.90 C_a_ − 63.14 C_b_)/214 (4)

where

C_a_ and C_b_ are the concentrations of chlorophyll a and chlorophyll b, respectively
A_662_, A_645_, A_470_ are absorbances at 662, 645, and 470 nm, respectively

The total concentrations of chlorophyll a, chlorophyll b, and carotenoids were then expressed as the total contents per unit of dry biomass weight (mg g^−1^).

### 2.5. Determination of Carotenoid Profiles Using APCI-QTOF MS/MS

The filtered microalgal extracts were subjected to APCI-QTOF MS/MS analysis using a SCIEX X500R QTOF mass spectrometer (Framingham, MA, USA). Liquid chromatography (LC) separation was conducted at 40 °C using a Synergi™ 4 µm Fusion-RP 80 Å LC column (50 × 2 mm) (Phenomenex, Torrance, CA, USA). The mobile phase consisted of solvent A (dichloromethane/ethanol/acetonitrile/water = 5:50:5.5:39.5, *v*/*v*) and solvent B (dichloromethane/methanol/acetonitrile/water = 25:28:42.5:4.5, *v*/*v*). The LC gradient was operated as follows: 10% B for 2 min, a linear gradient of 10–20% B for 5 min, and 70% B for 3 min. The injection volume was 10 µL and the flow rate was 0.5 mL min^−1^. The mass spectrometry (MS) conditions were as follows: ion source gas at 60 psi; drying gas temperature at 550 °C; positive mode; mass ranges of 100–1000 Da; declustering potential at 80 V; CAD gas flow rate at 7 L min^−1^; and ion spray voltage at 5500 V. The QTOF MS data were processed using the SCIEX OS 2.0.0 software with Information Dependent Acquisition (IDA) mode. The criteria for carotenoid identification in the LC-MS/MS analysis included an identical retention time, precursor m/z, MS^2^ fragment m/z, MS^2^ fragmentation pattern, and mass error, compared to the carotenoid standards. Compounds lacking a matching standard were literally compared to the previously published data using their corresponding retention time, precursor m/z, MS^2^ fragment m/z, and MS^2^ fragmentation patterns, as well as their maximum absorption spectra in the HPLC chromatogram [27,28,29,30].

### 2.6. Determination of Fatty Acid Profiles Using GC-FID Analysis

For the determination of fatty acid compositions and content, direct transesterification was performed as previously described by Zhu, S. et al. [31]. Fatty acid methyl esters (FAMEs) were obtained by incubating 20 mg of lyophilized cells in 2.5 mL of methanol containing 2% (*v*/*v*) H_2_SO_4_ at 80 °C for 2.5 h. After cooling of suspension, FAME extraction was carried out at room temperature using 1 mL of *n*-hexane and 1 mL of saturated NaCl. The upper *n*-hexane layer containing the FAMEs was collected for the analysis of fatty acid compositions and content using a GC-FID (Agilent 7890B Gas Chromatograph, Santa Clara, CA, USA). The injector was operated in split mode with a ratio of 5:1 and a split flow of 10.306 mL min^−1^ at 240 °C. The column used was a DB-23 column (60 m length × 0.25 mm i.d. × 0.25 µm film thickness) (Agilent, CA, USA). Helium was used as the carrier gas with a flow rate of 2 mL min^−1^, and 1 µL of FAME extract was injected. The initial temperature was at 50 °C for 1 min and increased to 175 °C at a rate at 25 °C min^−1^, then rose to 230 °C at a flow rate of 4 °C min^−1^ for 18 min. Individual FAMEs were identified by comparing them to the standard Supelco FAME 37 component mixture C4:0-C24:1 (Sigma-Aldrich), and the % fatty acid content was expressed as a relative percentage of the total fatty acids and calculated as previously described by Zhu, S. et al. [31]. Heptadecanoic acid was used as the internal standard.

### 2.7. Statistical Analysis

All experiments were performed in biological triplicates and the mean ± standard deviation from all data was analyzed using a statistical one-way ANOVA (GraphPad Prism 10.5.0). The post hoc Tukey’s multiple comparison test was used to test the differences among groups in different trials. The *p*-values of less than 0.05 were considered to be statistically significant.

## 3. Results

### 3.1. Effects of Chemical Modulators on C. zofingiensis Growth Profiles

**Figure 1:**
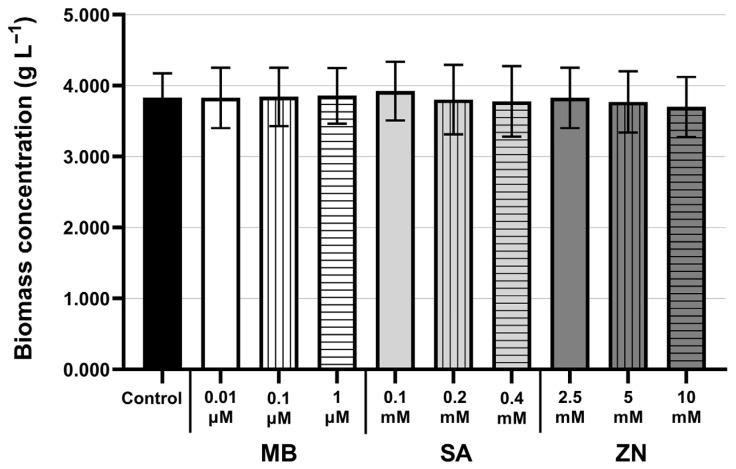
Biomass concentrations of control and cultures treated with different concentrations of chemical modulators measured at day 8 of cultivation. Treatments are grouped according to chemical modulator type; methylene blue (MB), salicylic acid (SA), and zinc sulfate heptahydrate (ZN). Chemical modulators were added at 96 h post-inoculation. All experiments were performed in biological triplicate, and values are expressed as mean ± SD.

Under mixotrophic cultivation, the *C. zofingiensis* cells rapidly exhibited cumulative growth prior to chemical treatment, as the specific growth rates of all *C. zofingiensis* cultures during the exponential phase were obtained ranging from 0.0217–0.0225 h^−1^ (Table S1). Prior to chemical treatment, similar growth curves among the mixotrophic *C. zofingiensis* cultures were detected (Figure S1). In the presence of MB, SA, and ZN, the biomass concentrations of all mixotrophic cultures at day 8 of cultivation were slightly changed, ranging from 3.700 ± 0.424 to 3.923 ± 0.415 g L^−1^, as compared to 3.831 ± 0.344 g L^−1^ in the control (Figure 1). However, all these values for biomass concentrations and growth curves were not statistically different from the control at *p* < 0.05. For the 1 µM MB treatment, there was a slight increase in biomass concentration (3.857 ± 0.390 g L^−1^) compared to the control. Among the SA treatments, the highest biomass concentration of 3.923 ± 0.415 g L^−1^ was obtained at 0.1 mM SA (Figure 1), higher concentrations of SA however caused a gradual decrease in biomass concentrations. Similarly, higher ZN concentrations lowered biomass concentrations. At 2.5 mM ZN, the *C. zofingiensis* biomass concentration was 3.828 ± 0.426 g L^−1^ and its increase to 10 mM gave a lowest value of 3.700 ± 0.424 g L^−1^ (Figure 1). Nevertheless, under the two-stage cultivation approach, these results indicated that *C. zofingiensis* could grow mixotrophically in various ranges of tested chemical concentrations. This suggested that the addition of these chemical modulators via a two-stage approach could trigger metabolite accumulation without impairing growth, thereby overcoming the growth inhibition typically associated with stress induction.

### 3.2. Effects of Chemical Modulators on Production of Astaxanthin, Lutein, and Canthaxanthin by HPLC Analysis

**Figure 2:**
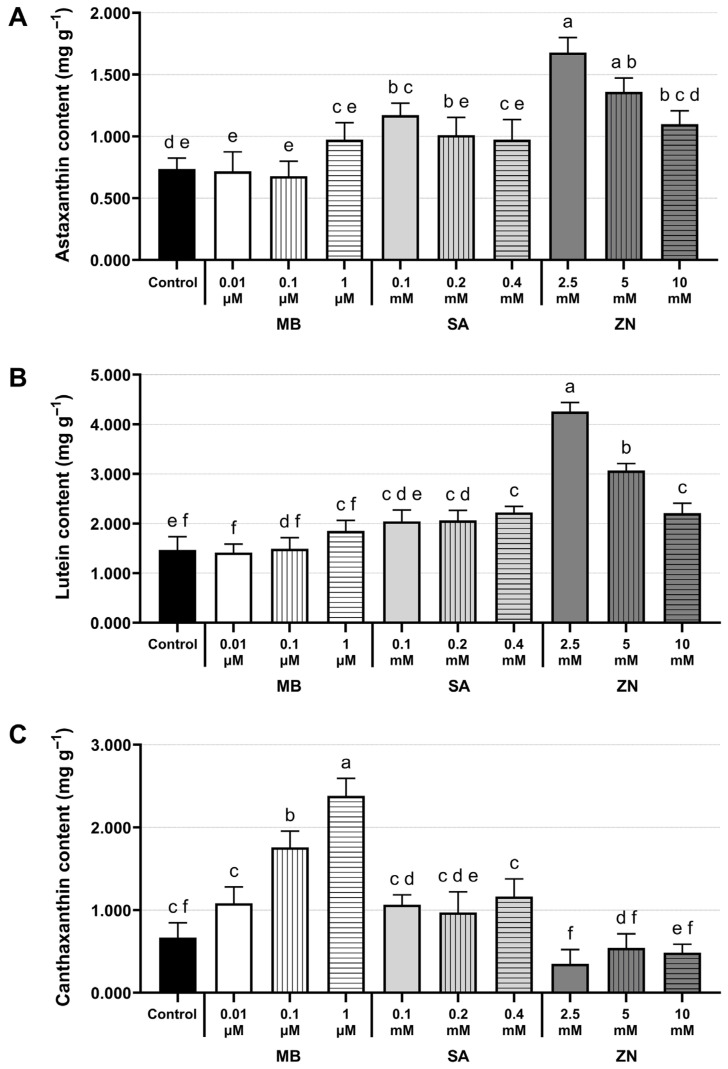
Pigment contents of astaxanthin, lutein, and canthaxanthin (**A**–**C**, respectively) in mixotrophic *C. zofingiensis* treated with different concentrations of MB, SA, and ZN. The different letters (a–f) indicate statistically differences in pigment contents at *p*-values < 0.05 (One-way Anova with Tukey’s test). All experiments were performed in biological triplicate, and values are expressed as mean ± SD.

To examine the pigment profiling and contents of astaxanthin, lutein, and canthaxanthin upon the chemical treatments, the 8-day-old lyophilized cells of the mixotrophic *C. zofingiensis* cultures treated with different concentrations of MB, SA, and ZN were extracted to obtain microalgal pigments. The target pigments, including astaxanthin, lutein, and canthaxanthin, were individually identified according to their retention times and maximum absorption spectra as compared to those of authentic carotenoid standards using HPLC analysis. Significant differences (at *p*-value < 0.05) in the induction effect of each chemical type and concentration on individual target pigments are indicated in Figure 2 and Supplementary Table S2 (see the different letter annotations).

**Figure 3:**
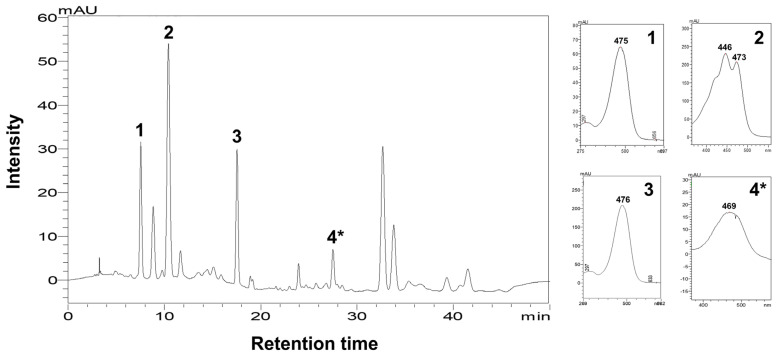
Representative HPLC chromatogram demonstrating pigments identified in *C. zofingiensis* culture with 2.5 mM ZN treatment. Peaks no. 1, 2, and 3 were identified as astaxanthin, lutein, and canthaxanthin, respectively. The inserted inlets exhibit maximum absorption spectrum of each of the identified carotenoid pigments; astaxanthin (1), lutein (2), and canthaxanthin (3). The inlet 4* shows the maximum absorption spectrum of peak no. 4* in the chromatogram, which was initially assigned as echinenone based on APCI-QTOF MS/MS data and subsequently confirmed by HPLC analysis with an authentic standard.

As compared to the control in Figure 2A, treatments of 0.1 mM SA, 2.5 mM ZN, and 5 mM ZN significantly increased astaxanthin contents. Among these treatments, ZN at 2.5 mM had the most stimulating effect on astaxanthin accumulation with the highest content of 1.679 ± 0.122 mg g^−1^, achieving a 2.28-fold increase over the control (Figure 2A). Similarly, *C. zofingiensis* treated with all ZN concentrations displayed a significant increase in lutein contents, for which the highest content of 4.257 ± 0.183 mg g^−1^ was obtained at 2.5 mM ZN treatment (a 2.91-fold increase over the control) (Figure 2B). In addition, 0.2 mM and 0.4 mM SA treatments also promoted lutein production in *C. zofingiensis* compared to the control (Figure 2B). Exposure to MB at 0.1 and 1 µM manifested canthaxanthin induction, with 1 µM MB exhibiting the highest canthaxanthin amount of 2.382 ± 0.210 mg g^−1^, which was 3.57-fold higher than the control (Figure 2C), while the addition of SA and ZN did not significantly enhance canthaxanthin production in *C. zofingiensis*. Interestingly, decreased amounts of canthaxanthin were observed in ZN-treated cultures (0.350 ± 0.173 to 0.542 ± 0.171 mg g^−1^) compared to the control (Figure 2C). Thus, the exogenous application of specific chemical modulators could differentially enhance targeted carotenoid accumulation in *C. zofingiensis* under a mixotrophic condition. The representative HPLC chromatogram exhibiting the identified carotenoid pigments and their maximum absorption spectra in the mixotrophic *C. zofingiensis* culture treated with 2.5 mM ZN is shown in Figure 3.

Correspondingly, as compared to the control, total contents of chlorophyll a and b were decreased in all treated *C. zofingiensis* samples, ranging from 6 to 77% reduction (Table S3). In contrast, total carotenoid content in *C. zofingiensis* across the various chemical modulator treatments increased by approximately 9–62%, and the highest total carotenoid content was observed in the 2.5 mM ZN treatment (Table S3).

### 3.3. Identification and Characterization of Carotenoid Compositions by APCI-QTOF MS/MS Analysis

To investigate additional carotenoid compositions produced in mixotrophic *C. zofingiensis* upon various chemical treatments, beyond those identified and validated by HPLC analysis, further pigment characterization was performed by APCI-QTOF MS/MS analysis. The identification of pigment compounds was achieved under the criteria of retention time, precursor *m*/*z*, MS^2^ fragment *m*/*z*, and MS^2^ fragmentation pattern, compared to the carotenoid standards and published data in the literature. As shown in Table 1 and Figure S2, the corresponding MS^2^ fragment ions of astaxanthin, lutein, and canthaxanthin, as ascribed in the literature, were found in our MS data, confirming the identification of these pigments, alongside the identification by HPLC analysis [27,28,29,30]. Notably, previously reported fragmentation ions used to characterize each carotenoid compound were detected in this study (see the bold numbers in Table 1), as some mass ions also provide validation for the presence of the associated functional groups in the corresponding carotenoid structures [27,28,29].

An additional pigment was detected in *C. zofingiensis* extracts, tentatively identified as echinenone based on APCI-QTOF data (Table 1 and Figure S2). The matched MS^2^ fragment ions of echinenone, including *m*/*z* 533.4158 and 551.4236, were detected in our mass spectrum, which is consistent with the previous findings [28,29], thereby supporting for the identification of this compound as echinenone (Table 1 and Figure S2). Moreover, the presence of this compound was further validated by comparison with an authentic echinenone standard (Sigma-Aldrich, St. Louis, MO, USA) using HPLC analysis. Echinenone was successfully identified in all microalgal crude extracts with a maximum absorption spectrum at 469 nm in the HPLC chromatogram (see peak and the inserted inlet no. 4* in Figure 3), the data of which are consistent with the prior report [27].

**Figure 4:**
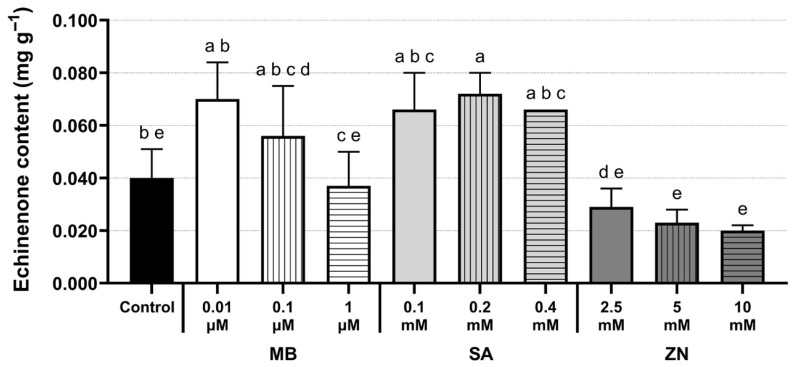
Echinenone contents in mixotrophic *C. zofingiensis* treated with different concentrations of MB, SA, and ZN. The different letters (a–e) indicate statistically differences in pigment contents at *p*-values < 0.05 (One-way Anova with Tukey’s test). All experiments were performed in biological triplicate, and values are expressed as mean ± SD.

The quantification of echinenone was also performed in this study. Its content was markedly lower than that of the three major pigments (astaxanthin, lutein, and canthaxanthin) across all control and treated *C. zofingiensis* cultures, ranging from 0.020 ± 0.002 to 0.072 ± 0.008 mg g^−1^ (Figure 4 and Table S2). Among all chemical treatments, the highest echinenone content of 0.072 ± 0.008 mg g^−1^ was detected in the SA treatment at 0.2 mM, representing an increase of approximately 1.8-fold compared to the control (0.040 ± 0.011 mg g^−1^) (Figure 4 and Table S2).

### 3.4. Effects of Chemical Modulators on C. zofingiensis Fatty Acid Compositions and Content

To determine the stimulatory effects of these chemical modulators on fatty acid production, the mixotrophic *C. zofingiensis* cultures treated with each chemical modulator type were extracted and transmethylated to obtain FAMEs for GC-FID analysis. The effects of each chemical at different concentrations on individual fatty acid compositions and their relative contents are presented in Table 2. The six major types of fatty acids identified herein were classified as the saturated fatty acids of palmitic acid (C16:0) and stearic acid (C18:0), the monounsaturated fatty acid of oleic acid (C18:1 n-9), and the polyunsaturated fatty acids of linoleic acid (C18:2 n-6), gamma-linolenic acid (C18:3 n-6), and alpha-linolenic acid (C18:3 n-3). Among these six fatty acids, the highest proportion of fatty acid profile obtained in all treated and control groups was for C18:1 n-9 followed by C16:0 (Table 2). Overall, the addition of MB, SA, and ZN altered the fatty acid profiles, and the individual contents of the six fatty acids showed minor fluctuations (either slight increases or decreases) across all chemical treatments when compared to the control. Nonetheless, statistical differences among these fatty acids were evident, as detailed in Table 2 and Figure S3. The C16:0 content was observed to be significantly decreased in the 0.01 µM MB, 1 µM MB, and 2.5 mM ZN treatments when compared to the control, where *C. zofingiensis*-treated with 2.5 mM ZN showed the lowest C16:0 content of 20.59%. Interestingly, the fatty acid contents of C18:0 were significantly decreased (by about 0.69-1.43%) across all chemical modulator treatments when compared to the control, except in the 0.1 mM SA and 2.5 mM ZN. The content of C18:1 n-9 was mostly increased in the MB and SA treatments, but a different result was observed for the ZN treatments. For the ZN treatment, a significantly improved accumulation of C18:1 n-9 by approximately 1.24% over the control was observed only at the 2.5 mM ZN concentration, but was found to be significantly decreased for 5- and 10 mM ZN treatments (Table 2 and Figure S3). In contrast, the content of C18:2 n-6 was increased together with the increasing ZN concentrations. The highest C18:2 n-6 content of about 16.24% was detected at 10 mM ZN, and was higher than the control by about 2.69% (Table 2 and Figure S3). Among the additions of MB, SA, and ZN, most treatments showed induction with only minor changes in the C18:3 n-6 content compared to the control, while a significant decrease was observed at 0.1 mM SA. For the C18:3 n-3 contents, treatment with increasing ZN concentrations resulted in a significant lessening of the C18:3 n-3 contents.

## 4. Discussion

In this study, cultivation of mixotrophic *C. zofingiensis* was performed by complying with a two-stage cultivation approach detailed in the Materials and Methods section. Among various sugars, glucose has been shown to be the best carbon source to promote algal cell growth [22]. Accordingly, our mixotrophic *C. zofingiensis* cultures in the presence of glucose exhibited rapid cell growth as high specific growth rates and biomass concentrations were obtained. Thus, the mixotrophic mode could offer an effective strategy to enhance high *C. zofingiensis* biomass production. The mixotrophic mode has also been favorably applied for culturing diverse microalgal species, such as *H. pluvialis, Chlorella sorokiniana*, and *Botryococcus braunii* [32,33,34]. Based on OD monitoring and specific growth rate analysis, at 96 h post-inoculation, we observed that the specific growth rate began to decline, indicating that the cultures were in the late exponential phase and were approaching the stationary phase. Therefore, in the present study, this time point was selected for the introduction of chemical modulators to implement a two-stage cultivation strategy, consisting of a biomass production phase (stage 1) followed by a metabolite induction phase (stage 2). Although residual glucose, nitrogen levels, and pH were not measured at the transition point, future studies should include these parameters to better provide additional insights into nutrient availability and the physiological status of *C. zofingiensis* before chemical application.

The concentration ranges applied in this study were defined based on previous reports for other algal species and preliminary screening under our laboratory conditions to achieve metabolite induction without severe growth inhibition. Our results showed that the application of MB, SA, and ZN to cells approaching the stationary phase did not markedly cause a severe cell death. Accordingly, the effective chemical concentrations used in this study are comparable with those reported for similar treatments in other microalgal species, including *H. pluvialis, C. vulgaris,* and *Crypthecodinium cohnii* [15,17,23,24]. Among the treatments, the highest biomass concentration of 3.923 ± 0.415 g L^−1^ was obtained at 0.1 mM SA (Figure 1). In a previous study, treatment with SA concentrations ranging from 2.5 to 15 mg L^−1^ in *C. vulgaris* 31 exhibited that a specific SA concentration (7.5 mg L^−1^) promoted cell growth, whereas the remaining tested concentrations generally resulted in lower algal growth than the control [23]. This is in compliance with the results in the present study that the addition of higher SA concentrations exhibited a trivial growth-promoting effect and a further increase in SA concentrations could suppress the growth of mixotrophic *C. zofingiensis* growth. Hence, the optimal concentration range for each modulator should be determined, as their growth-promoting efficacy is highly dependent upon the specific microalgal strain and environmental factors [23].

This study presents the influence of these specific chemical modulators (MB, SA, and ZN) via a two-stage cultivation approach on differentially stimulating the production of the three target carotenoid pigments in *C. zofingiensis*, including astaxanthin, lutein, and canthaxanthin. Previous studies have demonstrated the effectiveness of two-stage cultivation strategies in *C. zofingiensis* that enable biomass accumulation during the initial phase followed by the induction of astaxanthin or lipid production in the subsequent phase [35,36]. However, direct comparisons between one-stage and two-stage cultivation specifically involving chemical modulators in *C. zofingiensis* are limited. Therefore, the present study applied a two-stage strategy in which chemical modulators were exogenously introduced after sufficient biomass accumulation, aiming to minimize growth inhibition during early cultivation while promoting selective metabolite accumulation during the induction phase. Overall, following the chemical treatments in the second stage to induce pigment production, the total carotenoid contents increased significantly across all treated *C. zofingiensis* samples compared to the control, which is consistent with the results observed in the HPLC analysis. A simplified schematic of the carotenoid biosynthetic pathway representing the effects of specific chemical modulators and the major carotenoids identified in this study is provided in Supplementary Figure S4. The carotenoid biosynthetic pathway in *C. zofingiensis* utilizes a special route for astaxanthin synthesis, in which astaxanthin is primarily synthesized via the hydroxylation of β-carotene to zeaxanthin, catalyzed by β-carotene hydroxylase (CHYb), followed by the ketolation of zeaxanthin to astaxanthin catalyzed by β-carotene ketolase 1 (BKT1) (Figure S4) [37,38]. The ketolation of β-carotene catalyzed by the BKT1 enzyme produces the intermediate echinenone, which is subsequently converted to canthaxanthin [38]. In the MB-treated cultures, the canthaxanthin content increased proportionally with the MB concentration, suggesting a possible role for MB in regulating canthaxanthin accumulation in a dose-dependent manner (Figure 2). The application of MB at 1 µM could predominantly and significantly induce canthaxanthin accumulation, but it did not promote astaxanthin and lutein accumulation. Our results also revealed that MB had no induction effect on astaxanthin production, which is consistent with a previous study on *Chlorococcum* sp. [39], but distinct from *H. pluvialis*, in which MB primarily stimulated the production of astaxanthin [15]. Thus, the accumulation of specific carotenoids in response to chemical treatments appears to be species-specific in microalgae. Accordingly, alongside with its ability to induce carotenoid production, MB at the respective concentrations did not cause severe growth inhibition in *C. zofingiensis* under our tested conditions. A previous report has revealed that MB can undergo photodegradation without a photocatalyst under visible light radiation, with its degradation rate depending upon pH and the MB concentration [40]. Nevertheless, the rate of MB degradation and residual MB levels were not directly measured in this study and the precise duration of cells exposure to MB could be further determined using UV-visible spectroscopy in future studies. The assessment of residual chemical levels in harvested biomass or microalgal extracts is important for ensuring safety in downstream applications. Under the SA treatment in the present study, different SA concentrations selectively induced target pigments in *C. zofingiensis*, with 0.1 mM SA enhancing the astaxanthin content and 0.2–0.4 mM SA promoting lutein accumulation (Figure 2 and Table S2). The addition of SA in *H. pluvialis* also resulted in increased astaxanthin accumulation along with the upregulation of genes involved in carotenoid biosynthesis [17]. Moreover in *H. pluvialis*, lutein production decreased at low SA concentrations but increased at higher concentrations [18].

Noteworthy, the ZN treatment at 2.5 mM in *C. zofingiensis* was found to successfully enhance astaxanthin and lutein content by achieving an increase of 2.28- and 2.91-fold over the control, respectively, suggesting a dual stimulatory effect of ZN on the production of these two pigment contents (Figure S4). It was also observed that the elevated pigments content was inversely proportional to the concentration of ZN applied, demonstrating a concentration-dependent effect (Figure 2). Accordingly, in previous studies, the addition of different types of metal ions has been found to exert a positive effect on carotenoid accumulation in other microalgae, such as enhancing astaxanthin by Fe^2+^-EDTA in *H. pluvialis* [41,42]. Moreover, the combined use of oxidants and metal ions, including H_2_O_2_, NaClO, and Fe^2+^ ions, has also played a role in stimulating lutein accumulation in the microalga *C. protothecoides*, yielding a high lutein amount of up to 31.4 mg L^−1^ [43].

The APCI-QTOF MS/MS was used to validate the carotenoid pigments detected by HPLC analysis and to further identify additional pigments in response to chemical treatments. The observed characteristic fragmentation ions for astaxanthin, lutein, and canthaxanthin were in agreement with previously reported mass ions [27,28], providing strong validation of their identities. Our study employed the APCI ionization mode that has been a potent approach for carotenoid analysis and provides both positively charged and negatively charged molecular ions of carotenoids, resulting in high sensitivity [44]. Although experimental conditions (such as ionization mode, mobile phase, and instrument parameters) might be different between our analysis and previous studies, LC-MS/MS producing comparable data (matched mass ions) among studies can be a powerful method for microalgal carotenoid identification. Accordingly, highly accurate MS data might also provide complementary confirmation of those carotenoid identities to the HPLC results. Furthermore, the APCI-QTOF data in this study revealed the presence of echinenone, as its MS data was consistent with that reported in *C. zofingiensis* [28,29]. The presence of this compound was also successfully confirmed by HPLC analysis, with the highest echinenone content observed under the 0.2 mM SA treatment. In *C. zofingiensis,* echinenone serves as an intermediate in the carotenoid biosynthetic pathway (Figure S4), where its ketolation leads to the formation of canthaxanthin [14,37,38], thus its presence validates the carotenoid biosynthetic flow. Nevertheless, combined with the HPLC data, further identification of the remaining chromatographic peaks in the chromatograms among treatments would provide valuable insights into the carotenoid profiling in *C. zofingiensis* in response to chemical treatments.

In the present study, the carotenoid profiling data in *C. zofingiensis* revealed that the ZN treatment preferentially enhanced astaxanthin and lutein accumulation but did not significantly enhance canthaxanthin production. The MB treatment increased canthaxanthin accumulation whereas different SA concentrations selectively induced the accumulation of astaxanthin, lutein, and echinenone, as depicted in Figure S4. This suggested that MB, SA, and ZN may exert their influences on carotenoid accumulation through different physiological routes. MB, as an oxidant compound, may transiently affect the cellular oxidative stress response. SA may act as a stress-related signaling molecule. ZN is as an essential trace element that may influence metabolic activity and stress responses. Nevertheless, the roles of MB, SA, and ZN in modulating enzyme activity, metabolic flux, and the expression of key genes encoding enzymes in the carotenoid biosynthetic pathway were not directly investigated in this study and require further validation. To further examine the molecular mechanisms of pigment biosynthesis in response to chemical treatment in *C. zofingiensis*, qRT-PCR or transcriptome/metabolome analyses could be conducted to fully elucidate and provide an in-depth understanding of the pathway regulation and mechanisms responsible for pigment induction by each chemical modulator.

Based on the GC-FID analysis, treatments with MB, SA, and ZN altered the fatty acid profiles, with only minor variations in the relative levels of the six major fatty acids compared with the control. Among the fatty acids determined, the oleic acid (C18:1 n-9) was present in a predominant amount in *C. zofingiensis* cultured under mixotrophic conditions. A previous report also showed a high yield of C18:1 (of about 35.7%) being detected in heterotrophic *C. zofingiensis* grown in Kuhl medium supplemented with glucose [45]. We also observed that palmitic acid (C16:0) was the second most abundant fatty acid, following oleic acid. Thus, the elevated contents of oleic acid and palmitic acid in the mixotrophic *C. zofingiensis* cells observed in our study might contribute to its potential as a favorable host for producing high-quality biodiesel. Ideally, biodiesel requires fatty acid constituents that are both oxidatively stable and maintain stability at low temperatures [45]. Markedly, saturated fatty acids could contribute to oxidative stability, whereas unsaturated fatty acids might enhance low-temperature stability [46]. Beyond biofuel applications, these saturated and polyunsaturated fatty acids produced in microalgae are high-value compounds with significant commercialization potential in biorefinery applications [1]. Accordingly, in our study, the enhanced production of astaxanthin and lutein at the 2.5 mM ZN treatment was observed in compliance with the increased level of C18:1 n-9, suggesting that the ZN may selectively enhance carotenoid accumulation while altering the fatty acid profile of *C. zofingiensis* under the tested conditions. Increasing ZN concentration from 2.5 to 10 mM led to reduced C18:1 and increased C18:2 n-6 (linoleic acid), which might suggest the involvement of desaturase enzymes in this conversion [14]. Changes in fatty acid profiles may also be related to carotenoid accumulation, for example astaxanthin in *C. zofingiensis* has been shown to be associated with lipid droplets and is presented in esterified forms [8]. However, astaxanthin accumulation is not strictly dependent on de novo fatty acid biosynthesis in *C. zofingiensis* [38].

Despite the fact that target enhancement of carotenoid accumulation and altered fatty acid compositions were observed in our study, further investigations, including ROS quantification, assessment of residual chemical modulators, morphological and lipid droplet characterization, and molecular analyses, would be valuable for validating the proposed mechanisms underlying chemical modulator-mediated responses in *C. zofingiensis*. Taken together, favorable chemicals could be applied to microalgal cultures to achieve the elevated production of the desired microalgal compounds. Consequently, strategies that enable the production of multiple high-value bioproducts within a single cultivation cycle using common inducing factors are essential for achieving a maximum algal productivity, thereby ensuring economic feasibility in biorefinery and biodiesel applications.

## 5. Conclusions

A two-stage cultivation strategy was employed in our study to sequentially enhance microalgal biomass production in the first phase and induce the synthesis of their high-value bioproducts under stress conditions in the second phase. In this study, chemical modulators, including MB, SA, and ZN, triggered the accumulation of target carotenoids and altered the fatty acid compositions in mixotrophic *C. zofingiensis*. Various concentrations of each chemical modulator also differentially affected their contents and compositions. The present study revealed that this cultivation approach with the addition of selected chemicals in the second stage achieved significant metabolite induction (especially carotenoids) without the growth inhibition. Notably, ZN at a low concentration of 2.5 mM significantly induced the production of astaxanthin and lutein, which are considered as important compounds in the nutraceutical, pharmaceutical, and feed industries. The pigment contents of astaxanthin and lutein declined with increasing ZN concentration (5–10 mM), demonstrating a concentration-dependent effect. The addition of 1 µM MB significantly enhanced canthaxanthin. Different SA concentrations selectively promoted the accumulation of astaxanthin and lutein in *C. zofingiensis*. In addition, APCI-QTOF verified the carotenoid identities in accordance with the HPLC results. This approach also enabled the detection of echinenone, an intermediate in the carotenoid biosynthetic pathway. Combined with the HPLC analysis, echinenone was identified in the chromatogram, exhibiting the highest accumulation at 0.2 mM SA. The fatty acid compositions obtained in all treated and control cultures included saturated, monounsaturated, and polyunsaturated fatty acids, where the highest proportion of C18:1 n-9 was detected. In addition, C16:0 was found to be the second most abundant fatty acid in the profile. Specifically, treatment with 2.5 mM ZN produced the highest levels of C18:1 n-9 along with a high accumulation of astaxanthin and lutein, this suggests that ZN treatment at 2.5 mM could offer a favorable condition for enhancing target carotenoids (astaxanthin and lutein) and altering fatty acid compositions in *C. zofingiensis* cultures. Our findings provide the useful information on the potent chemical modulators are capable of promoting desired carotenoid and changes in fatty acid compositions in mixotrophic *C. zofingiensis* via a two-stage cultivation approach. This strategy could offer a practical framework to improve the economic viability of microalgal biorefinery applications.

## Figures and Tables

**Table 1:** Carotenoid pigments identified in mixotrophic *C. zofingiensis* grown under various chemical treatments using APCI-QTOF MS/MS analysis.

Compound	Retention Time (min)	Precursor *m*/*z* (Observed)	MS^2^ Fragment *m*/*z* (Observed)	Mass Error (ppm)	Ionization Type	Reference
Astaxanthin	5.27	597.3943	119.0861, **147.1167**, **201.1282**, **285.1859**, **379.2641**, **579.3839**, **597.4089**	0.9	[M + H]^+^	[28]
Lutein	5.75	568.4269	93.0707, 145.1021, 211.1494, 251.1807, **338.2613**, **430.3242**, **476.3649**, 568.4285	−1	[M]^+•^	[27,28]
Canthaxanthin	6.55	565.4035	93.0707, 145.1018, 133.0646, **203.1427**, 217.1595, 217.1595, **363.2678**, **413.2852**, 565.4024	−1	[M + H]^+^	[27]
Echinenone	5.64	551.4247	95.0862, 119.0861, 145.1018, 159.1176, 175.1487, 211.1494, 225.2593, 345.2593, 429.3171, **533.4158**, **551.4236**	0.3	[M + H]^+^	[28,29]

Bold numbers indicate the common MS^2^ fragment ions that are reported in the literature. Underlined numbers indicate highest % intensity of mass peak.

**Table 2:** Fatty acid compositions and content (%) identified in mixotrophic *C. zofingiensis* grown under different chemical treatments using GC-FID analysis.

Fatty Acid Composition and Content (%)	C16:0	C18:0	C18:1 n-9	C18:2 n-6	C18:3 n-6	C18:3 n-3
Control	22.46 ± 0.20 ^a,b^	7.43 ± 0.15 ^a^	48.77 ± 0.29 ^c^	13.55 ± 0.32 ^d,e^	6.40 ± 0.19 ^a^	1.38 ± 0.08 ^a,b^
0.01 µM MB	21.40 ± 0.27 ^d^	6.27 ± 0.18 ^c,d^	49.51 ± 0.20 ^a,b^	14.65 ± 0.21 ^c^	6.63 ± 0.09 ^a^	1.53 ± 0.08 ^a^
0.1 µM MB	22.24 ± 0.28 ^b,c^	6.37 ± 0.2 ^c,d^	49.52 ± 0.06 ^a,b^	13.89 ± 0.20 ^d^	6.48 ± 0.12 ^a^	1.50 ± 0.05 ^a^
1 µM MB	21.76 ± 0.20 ^c,d^	6.00 ± 0.13 ^d^	49.04 ± 0.18 ^b,c^	15.19 ± 0.13 ^b,c^	6.50 ± 0.12 ^a^	1.51 ± 0.06 ^a^
0.1 mM SA	22.41 ± 0.27 ^a,c^	7.06 ± 0.28 ^a,b^	49.64 ± 0.10 ^a^	13.65 ± 0.24 ^d,e^	5.89 ± 0.28 ^b^	1.35 ± 0.08 ^a,b,c^
0.2 mM SA	22.75 ± 0.24 ^a,b^	6.74 ± 0.30 ^b,c^	49.62 ± 0.11 ^a^	13.18 ± 0.21 ^e^	6.34 ± 0.16 ^a,b^	1.37 ± 0.10 ^a,b^
0.4 mM SA	22.45 ± 0.19 ^a,b^	6.01 ± 0.22 ^d^	48.89 ± 0.20 ^c^	14.68 ± 0.09 ^c^	6.58 ± 0.11 ^a^	1.39 ± 0.09 ^a,b^
2.5 mM ZN	20.59 ± 0.12 ^e^	7.09 ± 0.15 ^a,b^	50.01 ± 0.23 ^a^	14.74 ± 0.18 ^c^	6.37 ± 0.18 ^a^	1.21 ± 0.06 ^b,d^
5 mM ZN	23.04 ± 0.18 ^a^	6.19 ± 0.22 ^c,d^	47.46 ± 0.20 ^d^	15.52 ± 0.20 ^b^	6.65 ± 0.06 ^a^	1.14 ± 0.06 ^c,d^
10 mM ZN	22.91 ± 0.27 ^a^	6.45 ± 0.17 ^c,d^	46.72 ± 0.26 ^e^	16.24 ± 0.19 ^a^	6.58 ± 0.17 ^a^	1.10 ± 0.06 ^d^

The different letter annotations (a–e) in the same column indicate statistically differences between different treatment groups for each fatty acid content at *p*-values < 0.05 (one-way ANOVA with Tukey’s test). All experiments were performed in biological triplicate, and values are expressed as mean ± SD.

## Data Availability

All data are contained within the article.

## Supplementary Materials

The following supporting information can be downloaded at: https://www.mdpi.com/article/10.3390/life16050799/s1, Figure S1: Growth curves of control and treated mixotrophic *C. zofingiensis* cultures; Figure S2: Representative MS/MS mass spectra of target carotenoid pigments; Figure S3: Fatty acid compositions and relative contents (%) identified in mixotrophic *C. zofingiensis* treated with different concentrations of MB, SA, and ZN; Figure S4: A schematic representation of carotenoid biosynthetic pathway in *C. zofingiensis* and selective enhancement of astaxanthin, lutein, and canthaxanthin accumulation. Table S1: Specific growth rates of *C. zofingiensis* cultured under a mixotrophic condition prior to chemical treatment; Table S2: Pigment compositions and contents obtained from HPLC analysis in *C. zofingiensis* treated with different chemical modulators; Table S3: Total contents of chlorophyll a, chlorophyll b, and carotenoids in *C. zofingiensis* control and treated cultures.

## Abbreviations

The following abbreviations are used in this manuscript:

MB	Methylene blue
SA	Salicylic acid
ZN	Zinc sulfate heptahydrate
