# Supplementary material for: Differential Induction of Astaxanthin, Lutein, and Canthaxanthin with Altered Fatty Acid Profiles in Chromochloris zofingiensis via a Two-Stage Cultivation Approach Using Different Chemical Modulators
Source: Life (Basel). 2026 May 11;16(5):799. doi: 10.3390/life16050799 (PMC13208819; doi:10.3390/life16050799)
Supplement: Supplementary file 1 [file life-16-00799-s001.zip › 4_Figs suppl_Suthamat_Life 2026 05 05.pdf]

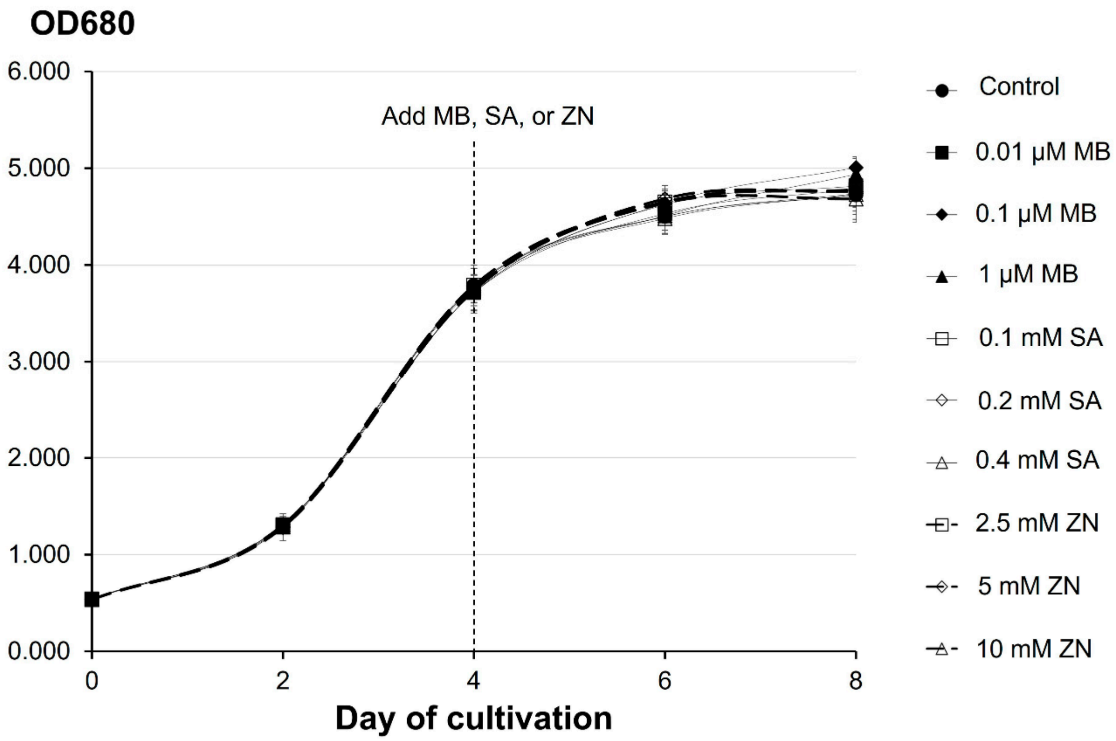

Figure S1. Growth curves of control and treated mixotrophic *C. zofingiensis* cultures. Algal growth in control and MB-, SA-, and ZN-treated cultures was monitored at OD680 every two days. All experiments were performed in biological triplicate, and values are expressed as mean ± SD.

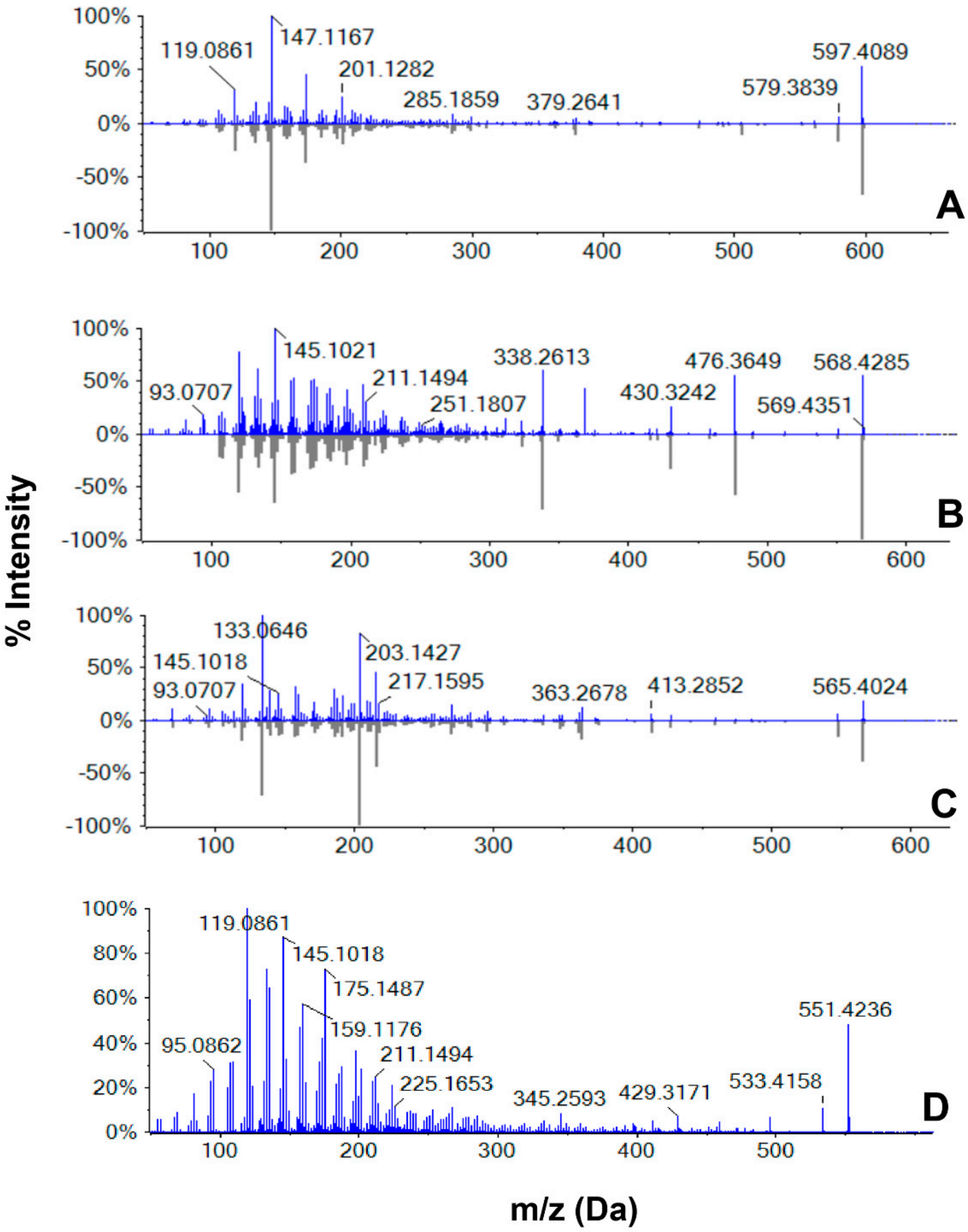

Figure S2. Representative MS/MS mass spectra of target carotenoid pigments. The mass spectra shown above 0% intensity line demonstrate MS^2 fragment m/z of astaxanthin (A), lutein (B), canthaxanthin (C), and echinenone (D) that were identified in mixotrophic *C. zofingiensis* crude carotenoid extracts using APCI-QTOF MS/MS analysis. The mass spectra below 0% intensity line shown in panel A, B, and C indicate MS/MS mass spectra of standard astaxanthin, lutein, and canthaxanthin, respectively.

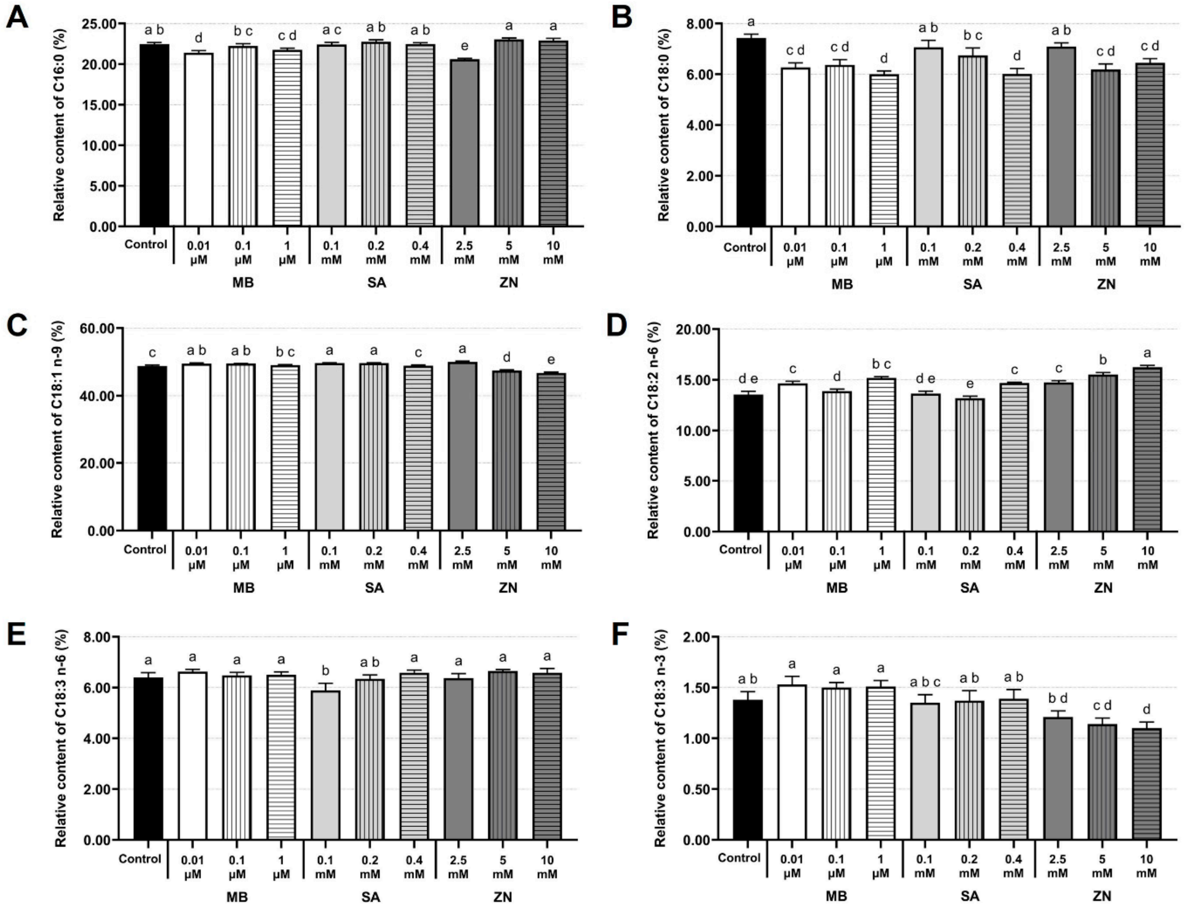

Figure S3. Fatty acid compositions and relative content (%) identified in mixotrophic *C. zofingiensis* treated with different concentrations of MB, SA, and ZN. Panel A-F represents relative fatty acid content of C16:0, C18:0, C18:1 n-9, C18:2 n-6, C18:3 n-6, and C18:3 n-3, respectively. The different letters (a-e) indicate statistically differences of fatty acid content at *p*-values < 0.05 (One-way Anova with Tukey's test). All experiments were performed in biological triplicate, and values are expressed as mean ± SD.

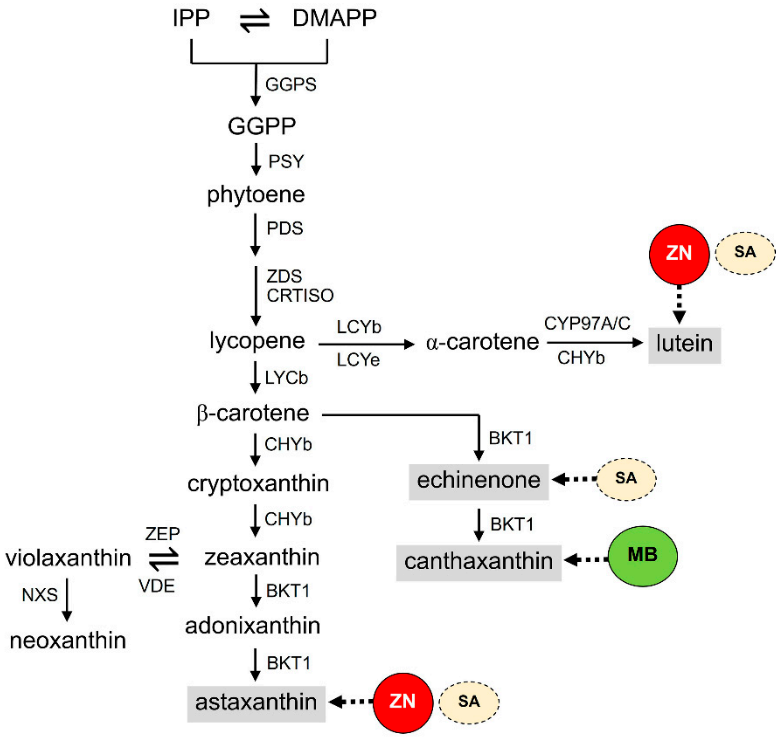

Figure S4. A schematic representation of carotenoid biosynthetic pathway in *C. zofingiensis* and selective enhancement of astaxanthin, lutein, and canthaxanthin accumulation. Among chemical treatments, ZN notably promoted astaxanthin and lutein accumulation, whereas MB mostly induced the production of canthaxanthin. Different SA concentrations selectively induced target pigments of astaxanthin, lutein and echinenone. The accumulation of these respective pigments varied depending on the chemical concentrations (see Figure 2 and Table S2).
