# Supplementary material for: Differential Induction of Astaxanthin, Lutein, and Canthaxanthin with Altered Fatty Acid Profiles in Chromochloris zofingiensis via a Two-Stage Cultivation Approach Using Different Chemical Modulators
Source: Life (Basel). 2026 May 11;16(5):799. doi: 10.3390/life16050799 (PMC13208819; doi:10.3390/life16050799)
Supplement: Supplementary file 1 [file life-16-00799-s001.zip › 5_Tables suppl_Suthamat_Life 2026 05 05.pdf]

Table S1. Specific growth rates of *C. zofingiensis* cultured under mixotrophic condition prior to chemical treatment.

Chemical type	Treatment	Specific growth rate (h^{-1})
**Control**	Control	0.0223
**Oxidant**	0.01 μM MB	0.0217
	0.1 μM MB	0.0217
	1 μM MB	0.0225
**Signal transducer**	0.1 mM SA	0.0220
	0.2 mM SA	0.0224
	0.4 mM SA	0.0220
**Metal ion**	2.5 mM ZN	0.0222
	5 mM ZN	0.0223
	10 mM ZN	0.0223

Table S2. Pigment compositions and contents obtained from HPLC analysis in *C. zofingiensis* treated with different chemical modulators.

**Chemical type**	**Treatment**	**Pigment content (mg g^{-1})**			
		**Astaxanthin**	**Lutein**	**Canthaxanthin**	**Echinenone**
**Control**	Control	0.737 ± 0.087 ^{d,e}	1.465 ± 0.270 ^{e,f}	0.667 ± 0.178 ^{c,f}	0.040 ± 0.011 ^{b,e}
**Oxidant**	0.01 µM MB	0.717 ± 0.158 ^{e}	1.415 ± 0.169 ^{f}	1.082 ± 0.198 ^{c}	0.070 ± 0.014 ^{a,b}
	0.1 µM MB	0.678 ± 0.122 ^{e}	1.492 ± 0.224 ^{d,f}	1.758 ± 0.197 ^{b}	0.056 ± 0.019 ^{a,b,c,d}
	1 µM MB	0.964 ± 0.137 ^{c,e}	1.851 ± 0.213 ^{c,f}	2.382 ± 0.210 ^{a}	0.037 ± 0.013 ^{c,e}
**Signal transducer**	0.1 mM SA	1.172 ± 0.097 ^{b,c}	2.041 ± 0.233 ^{c,d,e}	1.065 ± 0.119 ^{c,d}	0.066 ± 0.014 ^{a,b,c}
	0.2 mM SA	1.011 ± 0.143 ^{b,e}	2.060 ± 0.204 ^{c,d}	0.971 ± 0.251 ^{c,d,e}	0.072 ± 0.008 ^{a}
	0.4 mM SA	0.973 ± 0.163 ^{c,e}	2.223 ± 0.123 ^{c}	1.164 ± 0.214 ^{c}	0.066 ± 0.000 ^{a,b,c}
**Metal ion**	2.5 mM ZN	1.679 ± 0.122 ^{a}	4.257 ± 0.183 ^{a}	0.350 ± 0.173 ^{f}	0.029 ± 0.007 ^{d,e}
	5 mM ZN	1.361 ± 0.113 ^{a,b}	3.069 ± 0.142 ^{b}	0.542 ± 0.171 ^{d,f}	0.023 ± 0.005 ^{e}
	10 mM ZN	1.099 ± 0.108 ^{b,c,d}	2.207 ± 0.202 ^{c}	0.485 ± 0.102 ^{e,f}	0.020 ± 0.002 ^{e}

The different letter annotations (a-f) in the same column indicate statistically differences between different treatment groups for each pigment content at p-values < 0.05 (one-way ANOVA with Tukey's test). All experiments were performed in biological triplicate, and values are expressed as mean ± SD.

Table S3. Total contents of chlorophyll a, chlorophyll b, and carotenoids in *C. zofingiensis* control and treated cultures.

Treatment	Total content (mg g^{-1})		
	**Chlorophyll a**	**Chlorophyll b**	**Carotenoids**
Control	1.291 ± 0.016 ^{a}	0.778 ± 0.006 ^{a}	5.348 ± 0.048 ^{h}
0.01 µM MB	0.883 ± 0.030 ^{c}	0.489 ± 0.017 ^{c}	5.808 ± 0.028 ^{g}
0.1 µM MB	0.810 ± 0.026 ^{d}	0.457 ± 0.021 ^{c}	6.331 ± 0.072 ^{e}
1 µM MB	0.765 ± 0.013 ^{d}	0.457 ± 0.023 ^{c}	6.763 ± 0.097 ^{d}
0.1 mM SA	1.213 ± 0.040 ^{b}	0.658 ± 0.015 ^{b}	6.414 ± 0.067 ^{e}
0.2 mM SA	1.151 ± 0.003 ^{b}	0.653 ± 0.029 ^{b}	6.108 ± 0.040 ^{f}
0.4 mM SA	1.206 ± 0.026 ^{b}	0.660 ± 0.021 ^{b}	6.350 ± 0.052 ^{e}
2.5 mM ZN	0.298 ± 0.000 ^{f}	0.260 ± 0.000 ^{e}	8.658 ± 0.024 ^{a}
5 mM ZN	0.357 ± 0.016 ^{f}	0.303 ± 0.006 ^{d,e}	7.928 ± 0.013 ^{b}
10 mM ZN	0.455 ± 0.026 ^{e}	0.315 ± 0.021 ^{d}	7.155 ± 0.058 ^{c}

The different letter annotations (a-h) in the same column indicate statistically differences between different treatment groups for each pigment content at p-values < 0.05 (one-way ANOVA with Tukey's test). All experiments were performed in biological triplicate, and values are expressed as mean ± SD.
